# HCV Core Protein–ISX Axis Promotes Chronic Liver Disease Progression via Metabolic Remodeling and Immune Suppression

**DOI:** 10.1002/advs.202300644

**Published:** 2023-06-14

**Authors:** Li‐Ting Wang, Shen‐Nien Wang, Shyh‐Shin Chiou, Jhih‐Peng Tsai, Chee‐Yin Chai, Li‐Wen Tseng, Jin‐Ching Lee, Ming‐Hong Lin, Shau‐Ku Huang, Shih‐Hsien Hsu

**Affiliations:** ^1^ Department of Life Science National Taiwan Normal University Taipei 116059 Taiwan; ^2^ Center of Applied Genomics Kaohsiung Medical University Kaohsiung 80708 Taiwan; ^3^ Graduate Institute of Medicine College of Medicine Kaohsiung Medical University Kaohsiung 80708 Taiwan; ^4^ Division of General and Digestive Surgery Department of Surgery Kaohsiung Medical University Hospital Kaohsiung 80708 Taiwan; ^5^ Department of Surgery College of Medicine Kaohsiung Medical University Hospital Kaohsiung 80708 Taiwan; ^6^ Division of Pediatric Hematology and Oncology, Department of Pediatrics Kaohsiung Medical University Hospital Kaohsiung 80708 Taiwan; ^7^ Graduate Institute of Clinical Medicine, College of Medicine Kaohsiung Medical University Kaohsiung 80708 Taiwan; ^8^ Department of Pathology Kaohsiung Medical University Hospital Kaohsiung 80708 Taiwan; ^9^ Department of Biotechnology College of Life Science National Sun Yat‐sen University Kaohsiung 804201 Taiwan; ^10^ Department of Microbiology and Immunology School of Medicine College of Medicine Kaohsiung Medical University Kaohsiung City 80708 Taiwan; ^11^ Department of Medical Research Kaohsiung Medical University Hospital Kaohsiung Medical University Kaohsiung 80708 Taiwan; ^12^ National Institute of Environmental Health Sciences National Health Research Institutes Miaoli County 35053 Taiwan; ^13^ Department of Respirology & Allergy Third Affiliated Hospital of Shenzhen University Shenzhen 518020 China; ^14^ Department of Medicine Division of Allergy and Clinical Immunology Johns Hopkins University School of Medicine Baltimore MD 21287 USA

**Keywords:** HCV core protein, immune suppression, ISX, metabolic dysregulation, programmed death‐ligand 1 (PD‐L1)

## Abstract

Chronic hepatitis C virus (HCV) infection is an important public health issue. However, knowledge on how the virus remodels the metabolic and immune response toward hepatic pathologic environment is limited. The transcriptomic and multiple evidences reveal that the HCV core protein–intestine‐specific homeobox (ISX) axis promotes a spectrum of metabolic, fibrogenic, and immune modulators (e.g., kynurenine, PD‐L1, and B7‐2), regulating HCV‐infection relevant pathogenic phenotype in vitro and in vivo. In a transgenic mice model, the HCV core protein–ISX axis enhance metabolic disturbance (particularly lipid and glucose metabolism) and immune suppression, and finally, chronic liver fibrosis in a high‐fat diet (HFD)‐induced disease model. Mechanistically, cells with HCV JFH‐1 replicons upregulate ISX and, consequently, the expressions of metabolic, fibrosis progenitor, and immune modulators via core protein‐induced nuclear factor‐*κ*B signaling. Conversely, cells with specific ISX shRNAi inhibit HCV core protein‐induced metabolic disturbance and immune suppression. Clinically, the HCV core level is significantly correlated with ISX, IDOs, PD‐L1, and B7‐2 levels in HCC patients with HCV infection. Therefore, it highlights the significance of HCV core protein–ISX axis as an important mechanism in the development of HCV‐induced chronic liver disease and can be a specific therapeutic target clinically.

## Graphical abstract

Hepatitis C virus (HCV) core protein–intestine‐specific homeobox (ISX) axis promotes a spectrum of metabolic, fibrogenic, tryptophan catabolism enzymes, immune modulator (KYN, PD‐L1 and B7‐2), exacerbating HCV‐induced metabolic dysfunction, immune suppression, and fibrosis in vitro and in vivo. Mechanistically, HCV infection upregulates ISX and, consequently, the expressions of metabolic, fibrosis progenitor, and immune modulators (kynurenine, PD‐L1, and B7‐2) via core protein‐induced nuclear factor‐*κ*B signaling. ISX, Intestine‐specific homeobox; TRAFs, tumor necrosis factor receptor associated factors; KYN, kynurenine; MΦ, recruited macrophage, and Kupffer cells.

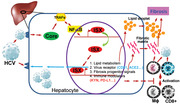

## 1 Introduction

Hepatitis C virus (HCV) infection is an important public health issue, with 1.9% (range: 1.5–2.3%) of the population infected worldwide.^[^ ^1^ ^]^ Approximately 30% (15–45%) of people with HCV infection can experience spontaneous clearance of the virus within 6 months of infection without treatment. However, 70% (55–85%) will develop chronic HCV infection, which progresses to chronic liver disease (cirrhosis, fibrosis, and hepatocellular carcinoma [HCC]) and metabolic disease (e.g., type II diabetes).^[^ ^1^, ^2^ ^]^ In 2016, ≈399 000 people died from HCV‐induced cirrhosis and HCC (primary liver cancer).^[^ ^2^ ^]^

Impaired metabolism and immune responses are key characteristics in chronic liver diseases including HCV infection.^[^ ^2^ ^]^ Patients with HCV infection, which is correlated with consequent metabolic disturbance and progresses to chronic liver disease, have high levels of serum immune suppression molecules (such as kynurenine and programmed cell death ligand 1 [PD‐L1]). Expression of several genome‐coded proteins, such as the core,^[^ ^3^ ^]^ NS3/4A,^[^ ^4^ ^]^ and NS5A,^[^ ^5^ ^]^ is correlated with the prognosis of HCV‐induced chronic liver disease. However, the mechanism underlying the association between HCV protein and the prognosis of chronic liver disease, immune suppression, and metabolic disturbance remains unclear. The core protein, one of the 10 HCV proteins, is an important oncogenic protein responsible for packaging viral RNA and virion budding.^[^ ^6^, ^7^ ^]^ Reduced sustained virologic response (SVR) is associated with a lower risk of decompensation cirrhosis and longer survival in HCC.^[^ ^2^ ^]^ Importantly, low SVR induced by direct‐acting antiviral (DAA) treatment does not completely reverse the effects of HCV infection on metabolic signals and the immune system.^[^ ^8^ ^]^

Intestine‐specific homeobox (ISX) is a homeobox‐containing protein that belongs to the paired subfamily and is homologous to Pax3, Pax7, and Prrx1 phylogenetically.^[^ ^9^ ^]^ Targeted *Isx* disruption in mice has revealed that ISX is required for the intestine‐specific regulation of high‐density lipoprotein receptors and cholesterol transporter scavenger receptor class B type 1 for vitamin A metabolism.^[^ ^9^ ^]^ ISX is induced by the pro‐inflammatory cytokine interleukin‐6 and highly expressed as a proto‐oncoprotein in hepatoma cell and HCC samples.^[^ ^10^ ^]^ Further, ISX transcriptionally regulates the downstream cell cycle proteins cyclin D1, E2F1,^[^ ^11^ ^]^ and indoleamine 2, 3‐dioxygenases, concomitant with dysregulated tryptophan catabolism and enhanced levels of immune checkpoint regulators (PD‐L1 and B7‐2) and epithelial–mesenchymal transition (EMT) regulators (Twist1 and Snail1), thereby affecting the survival time of patients with HCC.^[^ ^12^ ^]^ Pathologic studies revealed that ISX exhibits a tumor‐specific expression pattern, and that it is significantly correlated with patient survival and tumor size, number, and stage.^[^ ^13^ ^]^

The current study aimed to investigate the mechanism underlying the association between HCV infection, metabolic disturbance, and subsequent immune suppression as well as the development of chronic liver disease.

## 2 Results

### 2.1 HCV Core Protein Activates Metabolic and Preoncogenic Signals and Promotes Metabolic Disturbance and Fibrosis in Liver‐Specific Transgenic Mice Fed with High‐Fat Diet (HFD)

**Figure 1:**
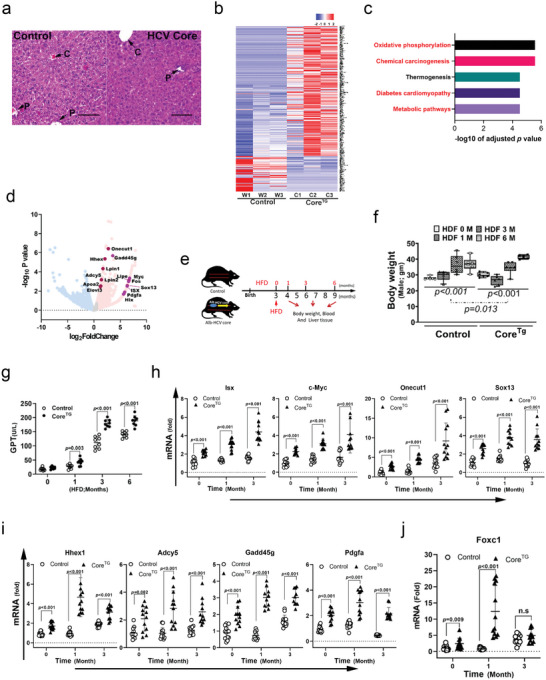
The hepatitis C virus (HCV) core activates intestine‐specific homeobox (ISX) expression in transgenic mice. a) Histological analysis of mouse liver via hematoxylin and eosin staining in HCV core transgenic and littermate control mice. b) Heatmap presentation of the major differentially expressed gene clusters in control and HCV core^TG^ mice. c) Major functional pathways of differentially regulated genes in HCV core^TG^ mice that were analyzed via Kyoto Encyclopedia of Genes and Genomes analysis. d) Volcano plot showing significant upregulation in red DEGs and significantly downregulation in blue DEGs. The genes of interest are specially labeled with larger circle. e) Scheme for high‐fat diet (60%) intake in HCV core transgenic and littermate control mice. f) Body weights measured in HCV core transgenic mice fed with a high‐fat diet (HFD) (*n* = 8). g) Representative images of control and HCV core^TG^ mice fed with HFD for 6 months. The levels of glutamic pyruvic transaminase in control and HCV core^TG^ mice with or without HFD for 6 months (*n* = 8). h–j) Relative mRNA levels of *Isx, c‐Myc, Onecut1, Sox13, Hhex1, Adcy5, Gadd45g, Pdgfa*, and *Foxc1* (*n* = 8). The results are shown as the mean ± s.d. Each experiment was repeated at least three times.

To validate the impact of the HCV core protein in vivo, liver‐specific core transgenic mice driven by albumin (*alb*) promoter^[^ ^14^ ^]^ were generated. At baseline without HFD and compared with wild‐type littermate controls, there were no differences in terms of body weight and size and serum levels of glutamic pyruvic transaminase (GPT), glutamic‐oxaloacetic transaminase (GOT), and triglyceride (TG) or in the histopathological analysis results between HCV core transgenic mice and littermates (**Figure** **1**a; and Figure S1a–c, Supporting Information). Since metabolic disturbance is an important pathogenic phenotype correlated with several metabolic human diseases and the development of hepatoma after HCV infection, the potential impact of HCV core protein on hepatic functional activity was evaluated in HCV core transgenic mice via next‐generation RNA sequencing. At baseline, transcriptomic analysis showed that 603 genes were significantly (*p* < 0.05) upregulated in HCV core transgenic mice, spanning a broad range of metabolic functions (Figure 1b,c). Pathway analysis on the Kyoto Encyclopedia of Genes and Genomes showed that, among the differentially expressed genes in HCV core transgenic mice, most pathways identified were associated with that in HCV infection. The top five pathways included signals of oxidative phosphorylation, signals of carcinogenesis (primarily proto‐oncogenes involved in HCC), thermogenesis, and signals of diabetes and metabolic pathways (primarily those in lipid and glucose metabolism) (Figure 1c). The 13 evidently highly expressed genes in HCV core transgenic mice included those involved in lipid metabolism (*Adcy5, Lpin1/2, Elovl3, Lipe*, and *Apoa2*), carcinogenesis (*Isx, c‐Myc, c‐Fos, Sox13*, and *Pdgfa*), and fibrosis progenitor signals (*Onecut1, Hhex1, Hlx*, and *Gadd45g*) (Figure 1d).

Then, core transgenic mice and littermates were challenged with HFDs (60%) for 6 months (Figure 1e). The body weight growth curves were slightly higher in male core transgenic mice fed with HFD for 6 months than in male littermates (*p* = 0.013) (Figure 1f). Although there were no significant differences in GOT levels between HCV core transgenic and littermate control mice, the GPT levels increased from the first month of HFD intake in HCV core transgenic mice compared with those in littermate control mice (Figure 1g). Other than *Lpin1/2* levels (Figure S1d and S1e, Supporting Information), the levels of the gene expression in lipid metabolism (*Adcy5* and *Lipe*), carcinogenesis *(Isx, c‐Myc, Sox13*, and *Pdgfa*), and the fibrosis progenitor signals (*Onecut1, Hhex1, Hlx*, and *Gadd45g*) in HCV core transgenic mice were significantly enhanced as compared to those seen in littermate controls after feeding with HFD. The expression levels) were higher in the HCV core transgenic mice than in control littermate mice (Figure 1h–j). The *Lpin1* level was significantly enhanced at baseline and at the third month after HFD feeding, but not at the first month after HFD feeding (Figure S1d, Supporting Information). Notably, the expression of hepatic Foxc1 showed differ at the first month after HFD feeding comparing HCV core transgenic mice with control littermates (Figure 1j).

### 2.2 HCV Core Protein Exacerbates Metabolic Disturbance and Liver Fibrosis in Transgenic Mice Fed with HFD

**Figure 2:**
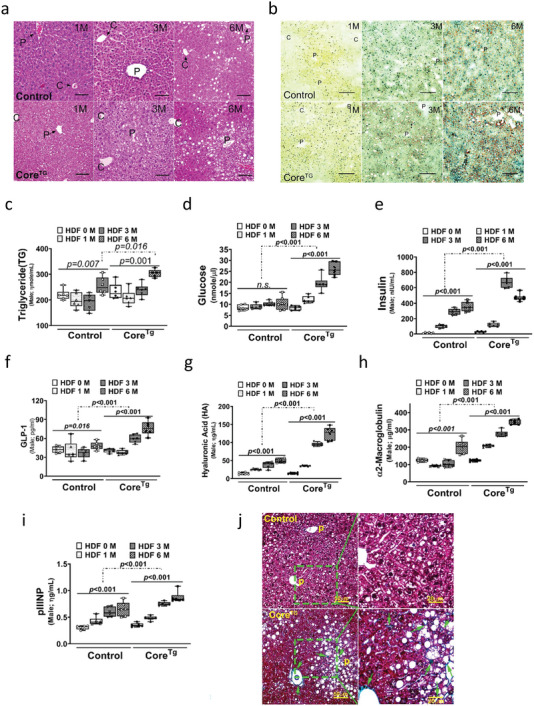
Hepatitis C virus (HCV) core–intestine‐specific homeobox (ISX) axis promotes lipid accumulation, liver fibrosis and insulin resistance in transgenic mice. C, central vein, and p, portal vein. Scale bar, 100 µm. a) Histological analysis of mouse liver examined via hematoxylin and eosin staining in HCV core transgenic mice fed with a high‐fat diet (HFD). Scale bar, 200 µm. b) The level of lipid droplet (orange) in the liver is stained with Oil Red O in HCV core transgenic mice fed with a high‐fat diet. Scale bar, 200 µm. c–e) Serum level of triglyceride (TG), glucose, and insulin detected in HCV core transgenic mice fed with HFD (*n* = 8). f–i) Serum level of glucagon‐like peptide 1, hyaluronic acid, *α*2‐macroglobulin, and pIIINP detected in HCV core transgenic mice fed with HFD (*n* = 8). j) Level of liver fibrosis (green) determined via Masson Trichrome staining in HCV core transgenic mice fed with HFD. The results are shown as the mean ± s.d. Each experiment was repeated at least three times.

Generally, histopathological analysis showed increased microvesicular steatosis, ballooning degeneration of hepatocytes, and minor scattered inflammation (mainly around the portal vein) in the livers of HCV core transgenic mice fed with HFD from the first month as compared with those noted in littermate controls. Further, macrovesicular steatosis, severe ballooning degeneration of hepatocytes, scattered inflammation, apoptotic bodies, and Mallory–Denk bodies (MDBs; around the portal vein) were observed in the livers of HCV core transgenic mice fed with HFD for 6 months (**Figure** **2**a). In terms of histopathological changes, control littermates fed with HFD for 6 months presented with minor macrovesicular steatosis with minimal or no ballooning degeneration of hepatocytes, scattered inflammation, apoptotic bodies, and MDBs (Figure 2a). The macrovesicular accumulated lipids were further detected via Oil Red O staining. There were more tiny lipid droplets (orange) in the livers of HCV core transgenic mice fed with HFD for 1 month. The size and number of these spots were remarkably increased relative to those of control littermates fed with HFD for 3 and 6 months (Figure 2b). The pathogenic outcomes of the HCV core protein on metabolic disturbance and liver fibrosis were monitored in transgenic mice with HFD. The serum levels of TG, glucose, and glucagon‐like peptide 1 (GLP‐1) in HCV core transgenic mice were significantly enhanced with HFD intake. However, the levels were slightly increased in control littermates (Figure 2c,d, and f). Interestingly, core transgenic and control mice presented with a continuous increase in serum insulin levels with HFD intake, and the core transgenic mice reached their highest serum insulin level after 3 months of HFD intake and then decreased (Figure 2e). In addition, serum hyaluronic acid (HA), which is an essential component of the extracellular matrix and is correlated with liver fibrosis, *α*‐2‐macroglobulin (A2M), and procollagen III amino terminal pro‐peptide (PIIINP), and serum biomarkers of liver fibrosis and fibrogenesis were used to evaluate the degree of liver fibrosis. HFD intake significantly enhanced the serum levels of HA, A2M, and PIIINP in HCV core transgenic mice. Meanwhile, HFD enhanced the expression of hepatic fibrosis markers in control littermates (Figure 2g–i). The extent of liver fibrosis was further verified via Masson's trichrome staining to monitor liver collagen accumulation. As shown in Figure 2j, a stronger signals of collagen accumulation (green, arrow) was detected around the portal (P) and central vein (C) in core transgenic mice fed with HFD for 6 months (Figure 2j). Further, collagen accumulation (green) was detected around ballooning degenerated hepatocytes of liver lobules in HFD‐fed core transgenic mice, but not in HFD‐fed controls (Figure 2j). There was no difference in metabolic functions (body weight, TG, glucose, insulin, and GLP‐1) and fibrosis markers (HA, A2M, and PIIINP) after HFD feeding between female HCV core transgenic mice and control littermates (Figure S2a–i, Supporting Information).

### 2.3 The HCV Core Protein Promotes ISX and Consequent Tryptophan Catabolism‐Relevant Immune Suppression Signals

**Figure 3:**
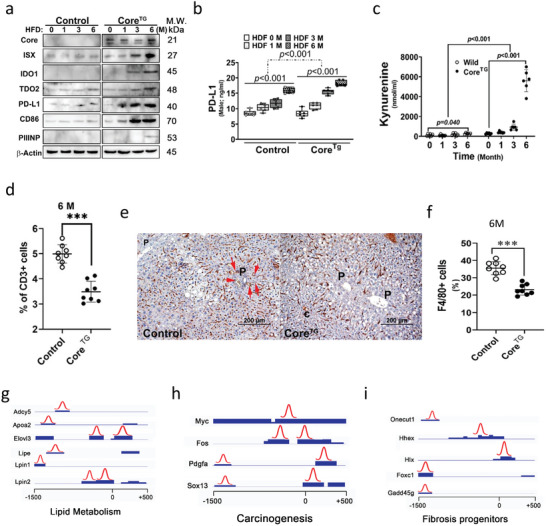
The hepatitis C virus (HCV) core–intestine‐specific homeobox (ISX) axis activates the serum levels of immune suppression regulators and decreases immune infiltration in transgenic mice. ***, *p* < 0.001. a) Protein expression levels of core, ISX, immune coinhibitors, and tryptophan catabolic enzymes were determined in HCV core transgenic mice fed with a high‐fat diet (HFD). b,c) The serum levels of PD‐L1 and kynurenine detected in HCV core transgenic mice fed with HFD (*n* = 8). d) Levels of infiltrated T cells in the liver stained with anti‐CD3^+^ antibody and counted via flow cytometry in HCV core transgenic mice fed with HFD (*n* = 8). e) Levels of liver macrophages (brown) determined via staining with anti‐F4/80 antibodies in HCV core transgenic mice fed with HFD. f) Levels of liver macrophages determined via flow cytometry in HCV core transgenic mice fed with HFD (*n*  =  8). g–i) The genomic binding fragments on gene promoters in g) lipid metabolism, h) carcinogenesis, and i) fibrosis progenitors and the peak calling of these analytes were all within 1 kbp of the promoter region. Results are presented as means ± s.d. Each experiment was repeated at least three times.

Of note, the HCV core protein significantly promoted ISX and consequent tryptophan catabolic enzymes (IDOs) and immune checkpoint molecules (PD‐L1 and CD86)^[^ ^13^ ^]^ at 0, 1, 3, and 6 months in transgenic mice although experienced minimal increases in the liver protein levels of ISX, IDOs, and immune checkpoint regulators (PD‐L1 and CD86) in control littermates fed with HFD (**Figure** **3**a). The protein level of PIIINP was increased in the core transgenic mice fed with HFD for 6 months, but not in control littermates fed with HFD (Figure 3a). The HCV core protein significantly upregulated the serum levels of PD‐L1 and a tryptophan metabolite, kynurenine (kyn), in both control littermates and core transgenic mice fed with HFD. Nevertheless, enhanced expression of serum kynurenine and PD‐L1 was more significant in core transgenic mice than that in control littermates (Figure 3b,c). Consequently, the HCV core protein significantly decreased the numbers of hepatic T cells (CD3+) (Figure 3d) and macrophages (F4/80+; brown) around the portal vein, particularly in transgenic mice compared with control littermates fed with HFD (Figure 3e,f). Furthermore, to gain potential molecular insights into the function of the proto‐oncogene‐*Isx* in controlling HCV core protein‐induced pathogenic progression, the target genes regulated by *Isx* in the livers of HCV core protein‐transgenic mice fed with HFD for 3 months were evaluated using chromatin immune precipitation sequencing with anti‐Isx MAbs. The peak calling of 150 bp genomic DNA fragments localized within the 1.5‐Kb promoter region of each target gene was considered *Isx* downstream targets. Of interest, a total of 1081 genes were targeted as positive Isx downstream genes, including genes involved in lipid metabolism (*Adcy5, Lpin1/2, Elovl3, Lipe*, and *Apoa2*), carcinogenesis (*c‐Myc, c‐Fos, Sox13*, and *Pdgfa*), and fibrosis progenitor signals (*Onecut1, Hhex1, Hlx*, and *Gadd45g*) (Figure 3g–i).

### 2.4 The HCV Core Protein Activates ISX and Consequent Tryptophan Catabolic Enzymes and Immune Coinhibitory Molecule Expression

**Figure 4:**
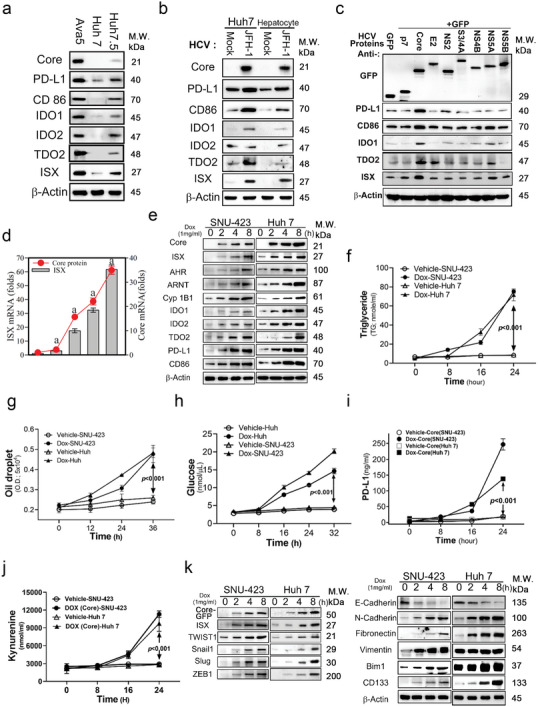
The hepatitis C virus (HCV) core activated the expression of intestine‐specific homeobox (ISX), immune coinhibitors, tryptophan catabolic enzymes, and EMT regulators in hepatoma cells. a) Protein expression levels of the HCV core, ISX, immune coinhibitors, and tryptophan catabolic enzymes determined in Ava5 (Huh 7 with HCV replicon), Huh 7 (hepatoma cell line), and Huh 7.5 (Ava5 without HCV replicon). b) Protein expression levels of the HCV core, ISX, immune coinhibitors, and tryptophan catabolic enzymes in Huh 7 and mouse hepatocytes transfected with mock or the JFH‐1 HCV genome. c) Protein expression levels of ISX, immune coinhibitors, and tryptophan catabolic enzymes in Huh 7 cells transfected with the component protein of HCV. d) The mRNA level of ISX was determined in SNU‐423cells with HCV core expression. e) Protein expression levels of the ISX–aryl hydrocarbon receptor axis, immune coinhibitors, and tryptophan catabolic enzymes in hepatoma cells with HCV core expression. f) The triglyceride level in culture media determined via enzyme‐linked immunosorbent assay (ELISA). g) The lipid droplet in Huh7 and SNU‐423 cells treated with doxycycline (1 *µ*g mL^−1^). h–j) The glucose, PD‐L1, and kynurenine levels in culture media determined via ELISA. k) The protein expression levels of ISX, EMT regulators (TWIST1, Snail, Slug, and ZEB1), mesenchymal (N‐cadherin, fibronectin, and vimentin), and epithelial markers (E‐cadherin) in hepatoma cells with HCV core expression were determined. The results are shown as the mean ± s.d. Each experiment was repeated at least three times.

To validate the potential impact of ISX in HCV core protein‐induced chronic liver disease, the HCV genomic (JFH‐1; genotype 2a) and core protein were transfected into hepatoma cells. First, the level of ISX in hepatoma cell lines, Ava‐5 (Huh‐7 harboring an HCV genotype 2a replicon), naïve Huh‐7, and Huh‐7.5 (Ava‐5 derivatized with no HCV replicon), was monitored. Ava‐5 and Huh‐7.5 had higher levels of ISX signals (IDOs, PD‐L1, and CD86) than naïve Huh‐7 cells (**Figure** **4**a). To validate the regulatory effect of HCV infection, Huh‐7 cells, and primary hepatocytes were transfected with HCV Japanese fulminant hepatitis (JFH)‐1 genomic RNA, and the levels of ISX and consequent tryptophan catabolic enzymes and immune checkpoint regulators were confirmed. The cells transfected with JFH1 genomic RNA had a high core protein expression of ISX, tryptophan metabolic enzymes (IDOs), and immune checkpoint regulators (PD‐L1 and CD86) (Figure 4b). The structural and nonstructural proteins from HCV genomics were then transfected into Huh‐7 cells to evaluate the activation effects on ISX and relevant signaling, respectively. Cells with core protein transfection had higher levels of ISX, IDOs, and immune checkpoint regulators than that of cells transfected with other HCV‐encoded proteins (Figure 4c). Cells with NS5A protein transfection had a minor activation effect on the levels of ISX and tryptophan metabolic enzymes (IDOs) (Figure 4c). Two hepatoma cells (Huh 7 and SNU‐423) with a doxycycline‐inducible system of GFP‐tagged HCV core protein were established to validate consequent signal induction. The cells had increased ISX expression in both mRNA and protein levels with Dox (doxycycline, 1 ng mL^−1^) treatment and IDOs and immune checkpoint regulators in the core‐GFP‐inducible expression system (Figure 4d,e). Cells with core protein expression had a time‐dependent enhancement in the medium levels of TG (oil droplet), glucose, kynurenine, and PD‐L1 (Figure 4f–j). In addition, cells with core protein expression presented with time‐dependent increase in the levels of epithelial–mesenchymal transition regulators (TWIST1, Snail1, Slug, and ZEB1) and mesenchymal cell markers (N‐cadherin, fibronectin, vimentin, BIM1, and CD133), but not epithelial marker (E‐cadherin) (Figure 4k). Notably, hepatoma cells with GFP‐core protein expression presented with enhanced mRNA and protein levels of virus infection‐relevant receptors, including CD36, CD81, Claudin1, LDL R, and ACE2 (Figure S3a,b, Supporting Information). In AVA5 cells, the HCV protein expression was significantly suppressed by treating cells with BILN‐2061 (a DAA for anti‐HCV 3/4A protease activity); however, the ISX expression was maintained, even slightly increased (Figure S3c, Supporting Information).

### 2.5 ISX Expression is Essential in HCV Core Protein‐Induced Metabolic Disturbance and Immune Coinhibitory Signals

**Figure 5:**
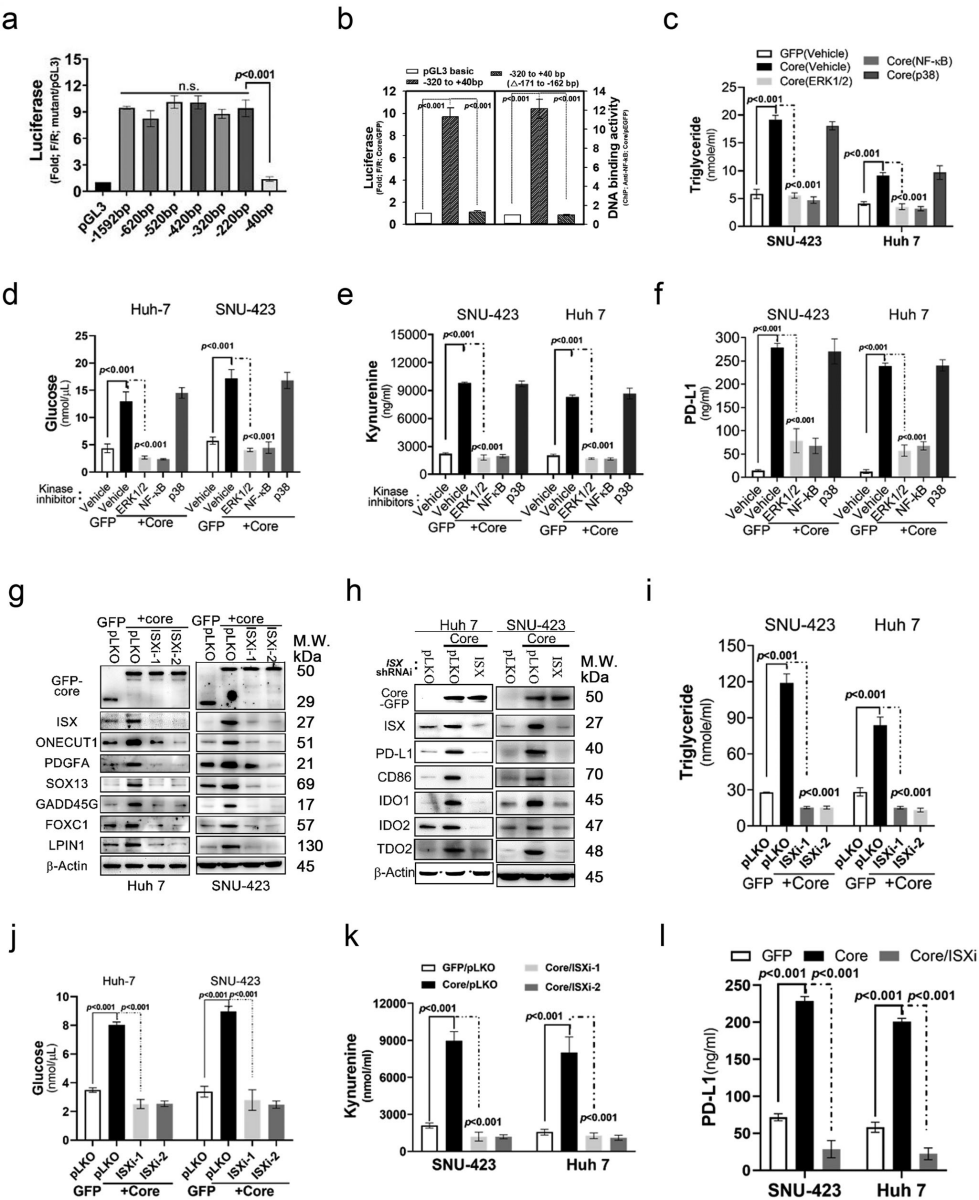
The intestine‐specific homeobox (ISX)‐mediated hepatitis C virus (HCV) core induced immune suppression signals via the cis transactivation of NF‐*κ*B. a,b) Transactivation effects of NF‐*κ*B induced by the HCV core on the ISX promoter verified via luciferase a) and chromatin immunoprecipitation b) assay. c–f) Levels of triglyceride, glucose, kynurenine, and PD‐L1 detected in the culture medium of hepatoma cells with HCV core expression and treated with or without protein kinase inhibitors. g,h) Protein expression levels of ISX, immune coinhibitors, and tryptophan catabolic enzymes determined in hepatoma cells with HCV core expression and transfected with or without ISX shRNAi. i–l) Levels of triglyceride, glucose, kynurenine, and PD‐L1 detected in the culture medium of hepatoma cells with HCV core expression with or without ISX shRNAi. The results are shown as the mean ± s.d. Each experiment was repeated at least three times.

To evaluate the impact of ISX in HCV core protein‐induced metabolic disturbance and immune suppression, cells with Dox‐induced core protein were treated with different inhibitors of the kinase signal pathways. Cells treated with the kinase inhibitors of MAPK (U0126, 5 ng mL^−1^) and NF‐*κ*B (BAY 11–7082, 20 ng mL^−1^) showed an abolish on the expression of relevant‐kinase, ISX and aryl hydrocarbon receptor (AHR)^[^ ^13^ ^]^ that were induced by forced core protein expression (Figure S3d, Supporting Information). Cells with forced core protein expression were then transfected with a Firefly luciferase expression construct driven by ISX promoter, and they presented with an enhanced ratio of Firefly/Renilla luciferase (internal control) activity until the ISX promoter was shorter than −220 bp (**Figure** **5**a). Cells with forced GFP‐core had activated promoter activity (ratio of Firefly/Renilla luciferase) driven by the ISX promoter. However, there was no effect on promoter activity driven by the ISX promoter with deletion between −162 and −171 bps (containing conserved NF‐*κ*B [p65] element) (Figure 5b, left). Cells with forced GFP‐core expression had a higher ISX promoter binding activity in an anti‐NF‐*κ*B (p65) chromatin immunoprecipitation (ChIP) assay, and no binding activity on ISX promoter was detected in the anti‐NF‐*κ*B (p65) ChIP assay transfected with the ISX promoter with a deletion between −162 and −171 bps (Figure 5b, right). The hepatoma cells (Huh 7 and SNU‐423) treated with NF‐*κ*B inhibitor inhibited the enhancements of TG, glucose, kynurenine, and PD‐L1 induced by the GFP‐core protein as well as MAPK inhibitor (U0126) treatment, an inhibitor of ISX phosphorylation^[^ ^15^ ^]^ (Figure 5c–f). Hepatoma cells (Huh 7 and SNU‐423) transfected with ISX shRNAi inhibited the enhancement of metabolic and fibrogenic progenitor genes and IDOs and immune checkpoint regulators induced by the HCV core protein (Figure 5g,h). Consequently, cells transfected with ISX shRNAi inhibited the enhancement of TG, glucose, kynurenine, and PD‐L1 levels induced by forced GFP‐core protein expression (Figure 5i–l).

### 2.6 ISX is Required for Immune Suppression Induced by the HCV Core Protein

**Figure 6:**
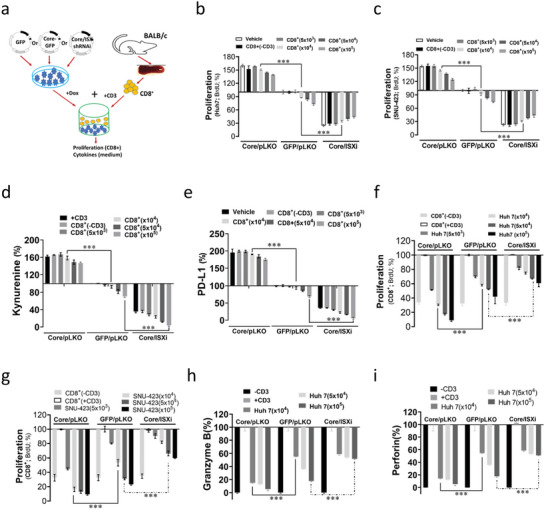
Intestine‐specific homeobox (ISX)‐mediated HCV core induced immune suppression effect on CD8^+^ T cells. ***, *p* < 0.001. a) Scheme for the coculture system of CD8+ T cells with hepatoma cells with HCV core expression with or without ISX shRNAi to evaluate immune suppression effects. b,c) Proliferation activities detected in Huh7 and SNU‐423 cells (×10^4^) with HCV core expression with or without ISX shRNAi cocultured with activated CD8+ T cells. d,e) Levels of kynurenine and PD‐L1 detected in the culture medium of hepatoma cells showing HCV core expression with or without ISX shRNAi cocultured with activated CD8+ T cells. f,g) Proliferation activities detected in activated CD8^+^ T cells (×10^4^) cocultured with SNU‐423 and Huh7 cells with HCV core expression with or without ISX shRNAi. h,i) Levels of granzyme B and perforin detected in the culture medium of activated CD8+ T cells (×10^4^) cocultured with hepatoma cells with HCV core expression with or without ISX shRNAi. The results are shown as the mean ± s.d. Each experiment was repeated at least three times.

To validate the immune suppression effects driven by the HCV core protein, hepatoma cells (Huh 7 and SNU‐423) with GFP or GFP‐core expression were individually cocultured with activated spleen CD8+ cells by anti‐CD3 antibody to evaluate the immune suppression effects in vitro (**Figure** **6**a). Cocultured hepatoma cells transfected with HCV core protein showed a significantly enhancement (Huh 7 (50%) and SNU‐423(45%)) in cell proliferation activity instead of a minor suppression (Huh 7 (12%) and SNU‐423 (9%)) in cocultured hepatoma cells with GFP only with (Figure 6b,c). Activated spleen CD8+ cells by anti‐CD3 antibody significantly inhibited the cell proliferation activity of hepatoma cells transfected with an ISX‐specific shRNAi construct (75% and 76%) compared with that of hepatoma cells with GFP only (Figure 6b,c). In addition, hepatoma cells transfected with the HCV core protein presented with significantly enhanced levels of kynurenine (52%) and PD‐L1 (83%). Meanwhile, hepatoma cells with GFP cocultured with activated spleen CD8+ cells alone by anti‐CD3 antibody had minor suppression in the levels of kynurenine (28%) and PD‐L1 (36%) (Figure 6c,d). Activated spleen CD8+ cells by anti‐CD3 antibody significantly inhibited the levels of kynurenine (85%) and PD‐L1 (92%) in hepatoma cells transfected with an ISX‐specific shRNAi construct compared with that of hepatoma cells treated with GFP along (Figure 6c,d). Reversely, hepatoma cells (Huh‐7 and SNU‐423) with HCV core expression presented with significantly suppressed cell proliferation activity ((Huh 7 (69%) and SNU‐423(83%)) of activated splenic CD8+ cells by anti‐CD3 antibody compared with GFP expression alone ((Huh 7 (58%) and SNU‐423(53%)) (Figure 6e,f). Hepatoma cells (Huh‐7 and SNU‐423) cotransfected with HCV core and ISX‐specific shRNAi had a minor suppression in the cell proliferation activity ((Huh 7 (33%) and SNU‐423(34%)) of activated splenic CD8+ cells by anti‐CD3 antibody compared with that of GFP expression alone (Figure 6e,f). Moreover, consistent with the results of cocultured with CD8+ T cells, hepatoma cells (Huh‐7) with HCV core expression presented significantly suppressed cell proliferation activity of activated splenic CD4+ cells by anti‐CD3 antibody compared with GFP expression alone or ISX‐specific shRNAi (Figure S3e, Supporting Information). Consequently, Huh 7 cells with forced GFP‐core expression presented with significantly suppressed level of granzyme B and perforin (94% and 94%, respectively) secreted by splenic CD8+ cells induced by anti‐CD3 antibody treatment compared with that of hepatoma cells cotransfected with ISX‐specific shRNAi construct (48% and 47%) or GFP (83% and 84%) alone (Figure 6g,h).

### 2.7 Severe HCV Infection is Associated with Poor Prognosis and Tryptophan Catabolic Enzymes and Immune Suppression Signals in Patients with HCC

**Figure 7:**
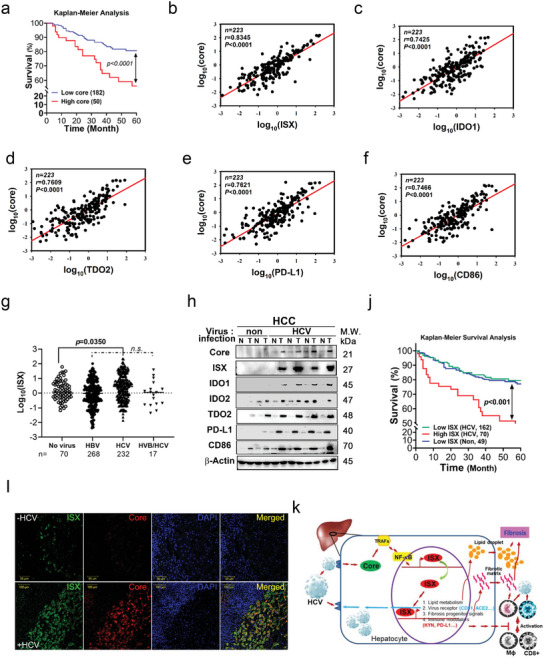
The hepatitis C virus (HCV) core was significantly correlated with immune coinhibitors and tryptophan catabolic enzymes and poor prognosis in patients with hepatocellular carcinoma (HCC). a) Kaplan–Meier survival curves of patients with HCV infection harboring low‐ and high core mRNA expressions who developed HCC. Based on the cutoff core mRNA value of a 3.5‐fold change relative to the neighboring normal tissue, the study population was divided into the high core (*n* = 182) and low core (*n* = 50) expression groups. b–f) HCV core mRNA expression was correlated with the mRNA levels of ISX, IDO1, TDO2, PD‐L1, and CD86 in HCC samples with HCV infection (*n* = 232). g) The mRNA levels of intestine‐specific homeobox (ISX) detected in patients without or with hepatitis virus infection who developed HCC. N.s., no significant difference. h) HCV core protein was coexpressed with ISX, PD‐L1, CD86, and IDOs (IDO1 and TDO2) proteins in tumor specimens collected from patients with HCC. i) Confocal immunofluorescent imaging of ISX and core in patients without or with hepatitis virus infection who developed HCC. j) Kaplan–Meier survival curves of patients with hepatocellular carcinoma with low ISX (no infection), low ISX (HCV infection), and high ISX (HCV infection) mRNA expressions. Based on the cutoff ISX mRNA value of a 3.0‐fold change relative to that in the neighboring normal tissue, the study population was divided into the low ISX (no infection, *n* = 49) and HCV patients with low ISX (*n* = 162) and high ISX (*n* = 70) expression groups. k) A schematic diagram illustrating the significance of the hypothetical model of HCV core–ISX axis in chronic liver disease prognosis in patients with HCV infection. Results are presented as means ± s.d. Each experiment was repeated at least three times.

To evaluate the effect of the HCV core–ISX axis on chronic liver disease, the mRNA levels of the HCV core protein, an HCV infection marker, and ISX signals were evaluated via a retrospective analysis of patients with HCC. The average survival duration after tumor resection was 60 months. Based on the assessment of 232 paired HCC and adjacent healthy tissue samples with HCV infection, patients with a high core mRNA expression (high group,″ *n* = 50) had a shorter survival than those with 1 low core mRNA expression (low group,″ *n* = 182) (*p* = 0.0006) (**Figure** **7**a). Notably, the core protein mRNA levels were significantly correlated with ISX in patients with HCV infection who developed HCC (Pearson, *r* = 0.8345, Figure 7b). The analysis of expression patterns in tryptophan catabolic markers and immune checkpoint regulators in tissue samples revealed significant correlations between core protein and the mRNA expression levels of PD‐L1, CD86, IDO1, and TDO2 in HCC tissue samples collected from patients with HCV infection (Pearson's correlation coefficient, *r* = 0.7621, 0.7466, 0.7425, and 0.7609, respectively; Figure 7c–f).

The mRNA levels of ISX in patients with HCV infection who developed HCC (*n* = 232) were significantly higher than in those without infection (*n* = 70), those with HBV (*n* = 268), or those with HBV/HCV (*n* = 17) (*p* = 0.0355, Figure 7g). The protein levels of ISX, tryptophan catabolic enzymes, and immune checkpoint regulators in tumor‐tissue parts and neighboring healthy parts in patients with or without HCV infection who developed HCC further determined. The core protein level of patients with HCV infection who developed HCC was more highly expressed in tumor tissues than in neighboring healthy tissues, concomitant with the protein levels of ISX, tryptophan catabolic enzymes, and immune checkpoint regulators (Figure 7h,i). As expected, the survival time was significantly shorter in HCV‐infected HCC patients with a high ISX mRNA expression (high group *n* = 70) than in HCV‐infected HCC patients with a low ISX mRNA expression (low group *n* = 162) or patients without HCV infection who developed HCC (*n* = 49) (*p* < 0.001) (Figure 7j). The baseline characteristics of patients with HCV infection who developed HCC were compared with those in other groups (Table S1, Supporting Information). There were significant differences among the high core/ISX, high core/low ISX, and the low core groups in terms of tumor size (*p* = 0.0259), number (*p* = 0.0218), and grade (*p* < 0.001) (Table S1, Supporting Information).

## 3 Discussion

This study showed that the HCV core protein transactivated proto‐oncogene‐ISX expression, resulting in metabolic dysregulation and immune suppression in vitro and in vivo. The HCV core–ISX axis promoted metabolic dysregulation (particularly in glucose and lipid) and immune suppression (tryptophan metabolite and immune checkpoint regulators, kynurenine, and PD‐L1), causing chronic liver disease progression in HCV core transgenic mice fed with HFD. Mechanistically, the HCV core transactivated ISX levels via the *cis* regulation of NF‐*κ*B (p65) on the ISX promoter element, which enhances the levels of ISX, tryptophan catabolic enzymes (IDOs), and immune checkpoint regulators (PD‐L1 and CD86 [B7‐2]), individually (Figure 7k). Reversely, cells with ISX deficiency caused by shRNAi inhibited metabolic disturbance and immune suppression induced by HCV core expression. Clinically, the HCV core level was significantly correlated with ISX, IDOs, PD‐L1, and CD86 levels in patients with HCV infection who developed HCC. Hence, the HCV core–ISX axis is important in chronic liver disease prognosis in patients with HCV infection.

Metabolic disturbance and impaired CD8+ T‐cell responses are the two major characteristics of HCV infection among different populations worldwide.^[^ ^6^, ^8^, ^16^ ^]^ Although most patients with HCV infection who are receiving DAA treatment have a high SVR, metabolic disturbance is still associated with a high risk of liver disease progression and cancer development after high SVR. The HCV core protein was considered one of the major candidates for metabolic disturbance and impaired CD8+ T‐cell responses as well as hepatic fibrosis and tumorigenesis.^[^ ^17^, ^18^ ^]^ The underlying mechanism remains unclear. The HCV core protein has been shown to activate NF‐*κ*B (p65) signals by interacting with tumor necrosis factor receptor‐associated factors involved in HCC progression.^[^ ^19^ ^]^ In the current study, the HCV core‐NF‐*κ*B signals have been shown to further transactivate ISX levels via the *cis* regulation of NF‐*κ*B (p65) on the ISX promoter element to initiate consequent pathologic progression. The HCV core–ISX axis transcriptionally activated a battery of genes in lipid and glucose metabolism, such as *Apos, c‐Myc, Onecut1, Elovl3, Adcy5, Foxc1*, and *Lpin1*, thereby providing a novel insight on the full range of HCV infection induced metabolic disturbance effects. Moreover, recent evidence indicated that kynurenine signals may cause the dysregulation of lipogenesis.^[^ ^20^ ^]^ Furthermore, elevation of the IL‐6 levels upregulated the hepatic ISX levels through Stat3‐NF‐*κ*B signals,^[^ ^13^ ^]^ thereby providing an alternative regulatory signal of ISX expression, and HCV infection is known to be associated with elevated serum IL‐6 levels.^[^ ^21^ ^]^

Patients with HCV infection have high levels of PD‐L1 and kynurenine and PD‐1^hi^ immune cells (including dendritic and CD8+ T cells), thereby resulting in HCV‐specific T‐cell exhaustion, immune suppression, and virus escape.^[^ ^22^ ^]^ Virus‐specific CD8+ cell dysfunction is an essential factor of persistent HCV infections which can be inhibited via the expression of coinhibitory molecules, such as the PD‐1/PD‐L1 axis and kynurenine.^[^ ^8^, ^18^ ^]^ Generally, CD8+ T‐cell dysfunction suppresses transcriptional reprogramming, broad metabolic alterations, and defective T‐cell effector function and memory development and the blockade of this signaling can allow the restoration of exhausted CD8+ T cells and increase the proliferation of HCV‐specific T cells.^[^ ^8^ ^]^ Additionally, the PD‐L1/PD‐1 axis has been shown to transfer macrophage polarization into the M2 subtype, thereby inhibiting macrophage phagocytosis and function.^[^ ^23^ ^]^ This study first showed that HCV infection induced the dysregulation of immune suppression via the HCV core–ISX axis in hepatocytes and hepatoma cells, which impairs CD8+ T‐cell functions and regulates the progression of consequent chronic liver disease. The ISX‐induced high PD‐L1 level was charged (at least partially) to the relevant immune suppression, including the impairment of CD8+ T‐cell functions and a decrease in the hepatic infiltration of T cells and macrophages. ISX transcriptionally regulated the downstream indoleamine 2, 3‐dioxygenases (IDOs), thereby dysregulating tryptophan catabolism and enhancing the levels of immune checkpoint regulators (PD‐L1 and B7‐1) and epithelial–mesenchymal transition (EMT) in patients with HCC.^[^ ^13^ ^]^ Notably, classic IFN‐*α* or DAA treatment did not inhibit enhanced HCV‐induced ISX‐relevant signaling affecting metabolic disturbance and immune suppression. This finding can provide a novel insight on improving the current treatment of HCV infection. Further, except immune suppression and metabolic disturbance, the HCV core protein also increased the expression of virus membrane receptors (*CD81, Claudin1, LDLR*, and *ACE2*), which increase the potential risk of HCV reinfection or coinfection with other viruses (such as HIV and COVID‐19).

Except for PD‐L1, the strict enhanced kynurenine levels induced by the core–ISX axis is another important determinant of immune suppression and chronic liver disease progression in an HCV core transgenic animal model. Kynurenine, a major bioactive metabolite in tryptophan catabolism, plays an important role in antimicrobial defense, immunosuppressive regulation, and disease tolerance.^[^ ^24^ ^]^ In patients with HCV infection, a high serum kynurenine level was detected in hepatocytes and DCs and was associated with liver metabolic dysregulation and cirrhosis.^[^ ^25^ ^]^ Kynurenine, a ligand of AHR, activates interleukin‐22 that modulates Treg differentiation and subsequent immune tolerance.^[^ ^26^ ^]^ Moreover, kynurenine–AHR signals were involved in the LPS‐Toll‐like receptor‐4 signals of Kupffer (macrophage) and DC cells, thereby contributing to the breakdown of endotoxin, activated macrophage polarization, and disease tolerance defense in liver fibrosis among patients with HCV infection.^[^ ^27^ ^]^

In conclusion, the HCV core activates ISX‐relevant signals causing metabolic disturbance and immunosuppression, which further promote liver fibrosis and insulin resistance in vitro and in vivo (illustrated in Figure 7k). Mechanistically, HCV infection upregulated ISX and, consequently, the metabolic, fibrosis progenitor, and immune modulators’ (kynurenine, PD‐L1, and B7‐2) expressions via core protein‐induced nuclear factor‐*κ*B signaling. Clinically, the HCV core is significantly correlated with the expression of ISX, IDOs, kynurenine, and PD‐L1 in patients with HCV infection who develop HCC. This emphasized the importance of the HCV core–ISX axis in the prognosis of HCV infection.

## 4 Experimental Section

### Plasmids

HCV full length genome (JFH‐1; genotype 2a) was a gift from Prof. Lee J. C. and full‐length HCV viral protein cDNA (including Core protein) was amplified using PCR from JFH‐1.^[^ ^28^ ^]^ The core protein cDNA was then subcloned into the *pEGFP/C1* vector (Clontech) to express a GFP‐tagged core protein. The *PLKO.1.puro* or *.neo* vector was used as a backbone for *shRNAi* constructs targeting *ISX*.

### Animal and Cell Culture

Core protein transgenic mice were obtained from the National Laboratory of Animal Breeding and Research Center (Taipei, Taiwan). All animal experiments were approved by the Kaohsiung Medical University–Institutional Animal Care and Use Committee (IACUC‐10181), Kaohsiung, Taiwan and performed accordioning with the guidelines of KMU Animal Care and Use Program for Center for Laboratory Animals, Kaohsiung Medical University, Taiwan. The animals were housing according to guidelines of the institution. The human liver cancer cell line, SNU‐423, was purchased from the American Type Culture Collection (ATCC; Manassas, VA) in May 2021. Ava‐5 (Huh‐7 harboring an HCV replicon), naïve Huh‐7, and Huh‐7.5 (Ava‐5 derivatized with no HCV replicon) cell lines, were obtained from the Bioresource Collection and Research Center (BCRC; Taipei, Taiwan) in July, 2020. Cell lines from both ATCC and BRC have been thoroughly tested and authenticated; morphology, karyotyping, and PCR‐based approaches were used to confirm the identity of the original cell lines. Cells were grown in 90% Eagle Minimum Essential Medium (MEM; Gibco, Grand Island, NY) with 2 mm L‐glutamine and Earle's Balanced Salt Solution (BSS; Gibco) adjusted to contain 1.5 g L^−1^ sodium bicarbonate, 0.1 mm nonessential amino acids (Gibco), 1.0 mm sodium pyruvate, and 10% fetal bovine serum (Gibco). All cell lines have been routinely tested for mycoplasma contamination using a Universal Mycoplasma Detection Kit (Thermo Fisher Scientific, Waltham, MA), and the last mycoplasma test was performed in December, 2021. Mycoplasma‐free cell lines were used in all experiments.

### Next‐Generation Sequencing (RNA‐Seq) and Data Analysis

Next‐generation sequencing was performed using the Illumina TrueSeq RNA Library Preparation Kit v2 with polyA selection to obtain 50 cycles of single‐end reads32. The reads were subsequently aligned to the March 2022 mouse reference sequence genome (GRCm38.p6) using the software Hisat2 (v2.0.1), after which the sample reads were visualized using the Integrated Genome Browser (Version 9.0.1). Next, differentially expressed genes were identified using the DESeq2 Bioconductor package. The DRDS function was used to calculate the false discovery rate (FDR) statistic for the significance of differentially expressed genes. Log‐transformed FPKM of >0.1 in at least one treatment group was used for the analysis. DEGs with fold changes greater than 1.5 and a log‐transformed FDR of 0.05 or less were only used. Means‐centered log‐transformed FPKM was used to prepare hierarchical clustering heatmaps in Cluster (version 3.0) and Java Tree View (version 3.0). A final significant differential gene list was used for gene enrichment analysis, including Gene Ontology (Biological Process) and the KEGG pathway.

### Primary Hepatocyte Isolation

Primary hepatocytes were isolated from Alb‐Cre mice and Alb‐Cre ISX loxP/loxP mice by liver dissociation kit (Miltenyi Biotec, Germany). The whole procedure was performed based on the guidelines of liver dissociation kit according to the manufacturer's instructions. Detailly, livers were dissected from mice and rinsed by PBS and cut into smaller pieces. Transferred livers into C tubes for gentleMACS dissociator and resuspended samples to allow flow through the cell strainers into 50 mL centrifuge tubes. After serious centrifugation, resuspended cells with cold PBS and debris removal solution (Miltenyi Biotec, Germany) carefully. Resuspended cells with low glucose DMEM, seeded into culture dishes. After 2 h the medium was changed to William's E medium (Gibco, USA) for further experiments.

### Western Blotting and Immunohistochemical Analysis

Western blotting and immunohistochemical (fluorescence) staining were performed as described previously.^[^ ^10^, ^29^ ^]^ The primary antibodies used in this study were PIINP and Actin polyclonal antibodies (1:5000 dilution; Sigma‐Aldrich), GFP monoclonal antibodies (1:500 dilution; Upstate, NY), FITC‐conjugated anti‐rabbit IgG, rhodamine‐conjugated antimouse IgG, and alkaline phosphatase‐conjugated anti‐rabbit IgG antibody (1:500 dilution; Jackson ImmunoResearch Laboratories), and CYP1B1 rabbit polyclonal antibody (1:200 dilution; Santa Cruz Biotechnology). The AHR goat polyclonal antibody (1:200 dilution; Santa Cruz Biotechnology). The Core protein, ISX, IDO1, TDO2, CD86, PD‐L1, N‐cadherin, E‐cadherin, Fibronectin, Vimentin, BMI1, CD133, TWIST1, Snail1, Slug, and ZEB1 (1:500 dilution) primary antibody was obtained from GeneTex International Corp. The Onecut1, GADD45G, PDGFA, SOX13, FOCX1, and LPIN1(1:500 dilution) primary antibody was obtained from Elabscience. All of the experiments were repeated at least 3 times.

### RNA Isolation and Real‐Time PCR

Total RNA from liver tissue RNA were isolated by RNeasy Mini Kit according to instructions from the manufacture (Qiagen, Valencia, CA) and then transcribed into cDNA (Invitrogen) for PCR amplification on a STEPONE Thermocycler (Applied Biosystems Inc.). Semiquantitative real‐time PCR was performed with SYBR Green FastMix (Applied Biosystem) and analyzed using ΔΔCt calculations. All data were normalized to GAPDH expression. All data are expressed as the mean ± SD of at least 3 experiments.

### Cell Proliferation Assay

A colorimetric immunoassay (Roche) for the quantification of the cell proliferation was performed based on the measurement of BrdU incorporation during DNA synthesis according to the manufacturer's instructions. The cells (10^4^) cultured in 96‐well plates were incubated at 37 °C for 16 h and then growth‐arrested prior to indicated treatment. The BrdU labeling reagent was added into the medium for 2 h incubation at 37 °C. Absorbance values were measured at 450 nm using a VersaMax ELISA Microplate Reader.

### Splenic CD8^+^ and CD4^+^ T Cell Isolation and Coculture Stimulation Conditions

Spleens were removed from Balb/c mice, and CD8^+^ and CD4^+^ T cells were isolated by positive selection with the use of magniSort CD8^+^ and CD4^+^microbeads according to the manufacturers protocol (eBioscience). SNU‐423 (Huh 7) cells and primary CD8+ T cells were cocultured in 96‐well plates for 24 h in RPMI medium. TCR stimulation consisting of 5 *µ*g mL^−1^ soluble anti‐CD3 (100 331, BioLegend).^[^ ^30^ ^]^ After 24 h, culture supernatant, hepatoma, and CD8^+^ (CD4^+^) cells were collected for analysis.

### ELISA Analysis

The levels of mouse GPT, triglyceride, glucose, GLP‐1, insulin, PD‐L1, Kynurenine, *α*2‐macroglobulin, hyaluronic acid, and pIIINP were determined by following the manufacturer's instructions on the ELISA kit from R&D Systems and Cusabio. First, blood was collected from the heart of a mouse and centrifuged. Then, serum was collected from the supernatant after centrifugation. Next, the serum was subjected to three repeated ELISA assays. All concentrations were calculated by referring to a standard curve of purified targets provided in the ELISA kit.

### Histological Analysis and Oil Red O Staining

Liver sections were fixed in 10% formalin overnight, embedded in paraffin, sectioned, and then stained with hematoxylin and eosin (H and E) for histological examination3. Masson's Trichrome staining (Poly Scientific, Bay Shore, NY) was performed according to the manufacturer's instructions provided on the kit, except for the step in which Aniline BlueSolution had to be added and incubated for 1–90 min. For oil red (ORO) staining of liver tissues, liver samples were embedded in an OCT compound for frozen sectioning and then stained with an ORO solution. The stained samples were placed under a microscope to acquire images at 200× magnification.^[^ ^31^ ^]^ The images were later quantified using the Image‐J software.

### Bioinformatics Analyses for the Expression Profiles

Gene ontology (GO) enrichment analysis and DAVID annotation were used for functional annotation and pathway analysis, such as for the molecular function, biological process, and cellular component. The Kyoto Encyclopedia of Genes and Genomes (KEGG, https://www.genome.jp/kegg/analyses) was consulted to evaluate the biological function of DEGs. GO terms with FDR (*p* < 0.05) were considered significantly enriched within the gene set.

### Patients

In this retrospective analysis, 232 patients (185 men and 47 women; mean age, 61.3 ± 5.89 years; range, 25–82 years) were included with confirmed HCC who had undergone curative hepatectomy between May 2017 to May 2022 from two medical centers (Chung Ho Memorial Hospital (148HCC), Taiwan Liver Cancer Network (50 HCC), and Changhua Christian Hospital (34 HCC)). No patient had undergone any preoperative treatment. The study was conducted with approval (KMUH‐IRB‐20180052 and KMUHIRB‐20130052) from the ethics committee of Kaohsiung Medical University Chung‐Ho Memorial Hospital. Written informed consent was obtained from each patient. The pathologic diagnosis and classification of variables were based on the criteria recommended in the General Rules for Clinical and Pathological Study of Primary Liver Cancer.^[^ ^32^ ^]^ Clinicopathologic characteristics collected for analyses included sex; age; levels of GOT, GPT, albumin, *α*‐fetoprotein, and Bilit and BCLC; tumor stage, size, and number. Tissue specimens, obtained during the surgery, were immediately stored in liquid nitrogen until further analysis. A group of patients with HCC was dichotomized into the “high‐” and “low”‐ISX or core protein expression subgroups according to the survival receiver operator characteristic (ROC) curve analysis.^[^ ^10^ ^]^ Hepatocellular carcinoma patients were classified into two groups‐ “low” and “high” according to survival receiver–operator characteristic (ROC) curve analysis. The cutting points of Core and ISX separately were 3.5 and 3.0 times of the mRNA expression in HCC tumors than that of the neighboring healthy tissues.SD, standard deviation. Statistical analysis of categorical variables were carried out by one‐way ANOVA; *, *p* < 0.05.

### Statistical Analysis

The quantitative variables are presented as the mean ± standard deviation (SD). Significant differences were determined using a two‐sample *t*‐test. Pearson's correlational analysis was used to examine the relationship between the levels of *ISX*, *IDO1*, *CD86*, and *PD‐L1* expression. Statistical analysis of categorical variables was performed using *χ* ^2^ analysis, one‐way analysis of variance, and Fisher's exact analysis. Differences with a *p* value < 0.05 were considered to be significant.

## Conflict of Interest

The authors declare no conflict of interest.

## Author Contributions

Conception and design: S.‐H.H. and L.‐T.W. Development of methodology: S.‐H.H. and L.‐T.W. Acquisition of data (provided animals, acquired and managed patients, provided facilities, etc.): L.‐T.W., C.‐Y.C., L.‐W.T., M.‐H.L., and S.‐N.W. Analysis and interpretation of data (e.g., statistical analysis, biostatistics, computational analysis): S.‐H.H., L.‐T.W., J.‐P.T., J.‐C.L. and S.‐N.W. Writing, review, and/or revision of the manuscript: S.‐H.H., L.‐T.W., S.‐K.H. Administrative, technical, or material support (i.e., reporting or organizing data, constructing databases): S.‐H.H. and S.‐S.C. Study supervision: S.‐H.H. and L.‐T.W.

## Supporting information

Supporting Information

## Data Availability

The data that support the findings of this study are available from the corresponding author upon reasonable request.
