# Supplementary material for: HCV Core Protein–ISX Axis Promotes Chronic Liver Disease Progression via Metabolic Remodeling and Immune Suppression
Source: Adv Sci (Weinh). 2023 Jun 14;10(23):2300644. doi: 10.1002/advs.202300644 (PMC10427408; doi:10.1002/advs.202300644)
Supplement: Supplementary file 1 — Supporting Information [file ADVS-10-2300644-s001.pdf]

ADVANCED SCIENCE
Open Access

Supporting Information

for *Adv. Sci.*, DOI 10.1002/advs.202300644

HCV Core Protein–ISX Axis Promotes Chronic Liver Disease Progression via Metabolic Remodeling and Immune Suppression

*Li-Ting Wang, Shen-Nien Wang, Shyh-Shin Chiou, Jhih-Peng Tsai, Chee-Yin Chai, Li-Wen Tseng, Jin-Ching Lee, Ming-Hong Lin, Shau-Ku Huang and Shih-Hsien Hsu**

Figure S1

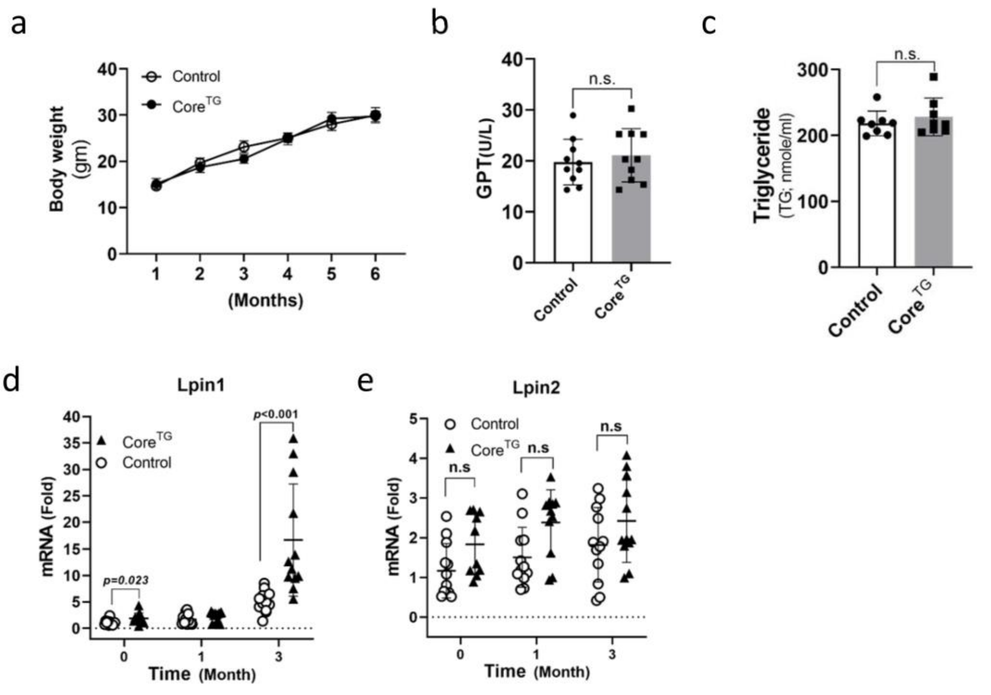

Figure S1. (a) Body weight gains in control (n = 8) and hepatitis C virus (HCV) core transgenic mice (n = 8). (b–c) Serum levels of glutamic pyruvic transaminase and triglycerides in control (n = 8) and HCV core transgenic mice (n = 8) ($p < 0.01$). (d-e) Relative mRNA levels of Lpin1 and Lpin2.

Figure S2

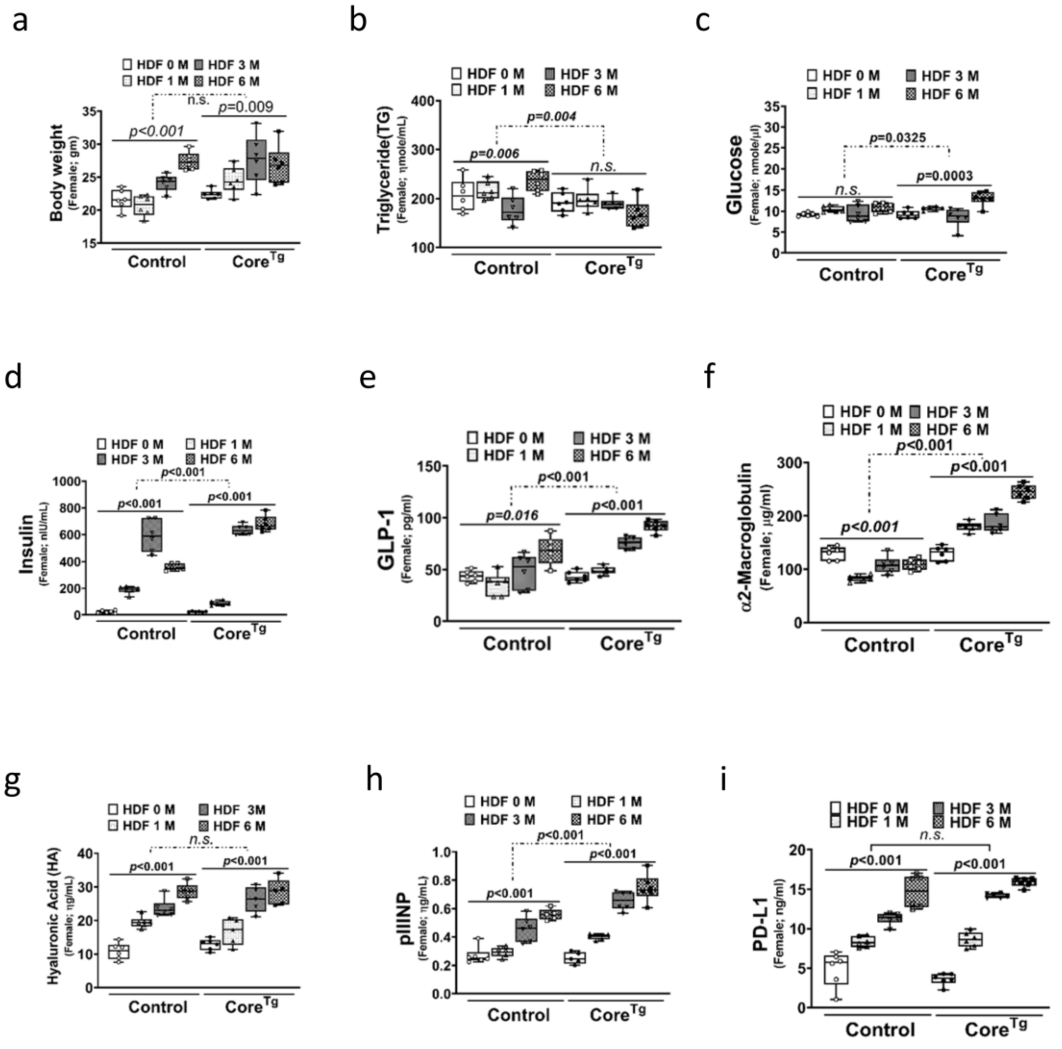

Figure S2. (a) Body weights measured in hepatitis C virus core transgenic mice fed with high-fat diet. Serum levels of triglyceride (b), glucose (c), insulin (d), glucagon-like peptide 1 (e), a2-macroglobulin (f), hyaluronic acid (g), procollagen III amino terminal pro-peptide (h), and programmed death ligand 1 (i) in female hepatitis C virus core transgenic mice fed with a high-fat diet. The results are shown as the mean ± s.d. Each experiment was repeated at least three times (n=8).

Figure S3

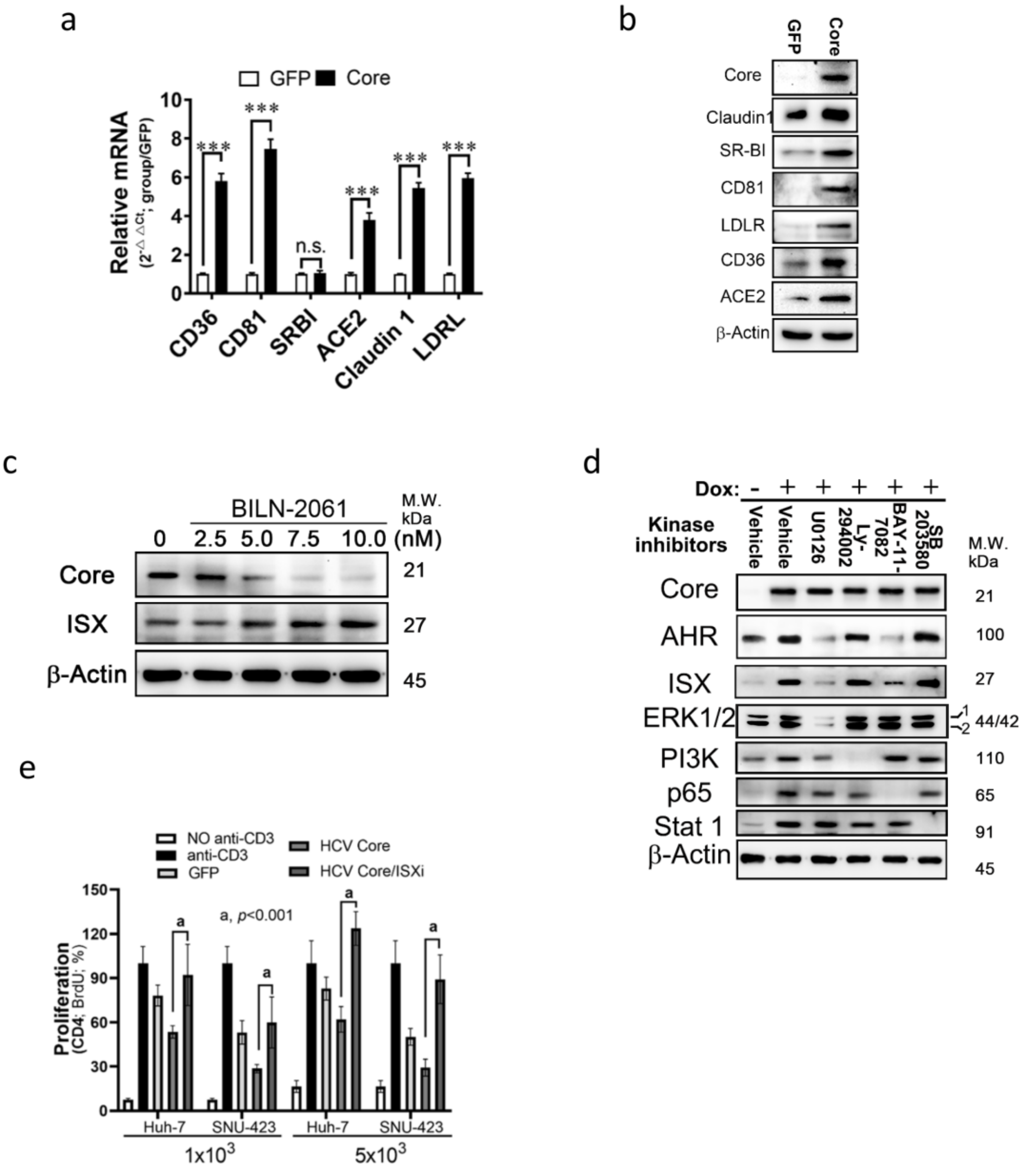

Figure S3. (a) The mRNA levels of membrane receptors (Claudin-1, CD36, CD81, SR-BI, LDL R, and ACE2) detected in Huh 7 cells with or without hepatitis C virus (HCV) core expression. ***, $p < 0.001$ and n.s., non-significant difference. (b) Protein levels of membrane receptors (CD36, CD81, SR-BI, Claudin 1, LDL R, and ACE2) in Huh 7 cells with or without HCV core expression. (c) Protein level of intestine-specific homeobox (ISX) detected in Ava5 cells treated with BILN-2061 (HCV NS3/4A inhibitor) individually. (d) Levels of ISX–aryl hydrocarbon receptor signals in

hepatoma cells treated with vehicle or different kinase inhibitors, individually. (e) Proliferation activities were detected in activated $CD4^+$ T cells ($\times 10^4$) co-cultured with Huh7 cells with HCV core expression with or without ISX shRNAi. Results are presented as means ± s.d. Each experiment was repeated at least three times.

Table S1. Baseline characteristics of 232 hepatocellular carcinoma (HCC) patients with HCV infection

**Group**	***Core Low*** **(n=182)(%)**	***Core*** **high/ISX low (n=8)(%)**	***Core*** **high/ISX high (n=42)(%)**	***p-value***
**GOT (U/L)**<br>**< 40**<br>**40≦ < 100**<br>**100≦**	**58(31.52)**<br>**91(49.46)**<br>**35(19.02)**	**4(50.00)**<br>**0(0)**<br>**4(50.00)**	**9(22.50)**<br>**16(40.00)**<br>**15(37.50)**	**0.1730**[#]
**GPT (U/L)**<br>**< 40**<br>**40≦ < 100**<br>**100≦**	**85(46.20)**<br>**73(39.67)**<br>**26(14.13)**	**3(37.50)**<br>**3(37.50)**<br>**2(25.00)**	**17(42.50)**<br>**18(45.00)**<br>**5(12.50)**	**0.1654**[#]
**Albumin (mg/dL)**<br>**< 4.5**<br>**≧4.5**	**172(93.48)**<br>**12(6.52)**	**8(100)**<br>**0(0)**	**40(100)**<br>**0(0)**	**0.7718**[#]
**α-Fetoprotein (ng/mL)**<br>**< 20**<br>**≧20**	**107(58.15)**<br>**77(41.85)**	**4(50)**<br>**4(50)**	**22(55.00)**<br>**18(45.00)**	**0.8519**[#]
**Bilit**<br>**1.5 <**<br>**≧1.5**	**158(85.87)**<br>**26(14.13)**	**8(100)**<br>**0(0)**	**39(97.50)**<br>**1(2.50)**	**0.1997**[#]
**Size(cm)**<br>**<2.5**<br>**2.5≦**	**70(38.04)**<br>**114(61.96)**	**2(25)**<br>**6(75)**	**6(15.00)**<br>**34(85.00)**	**0.0259***
**Number of tumors**<br>**1**<br>**1<**	**155(84.24)**<br>**29(15.76)**	**5(62.50)**<br>**3(37.50)**	**24(60.00)**<br>**16(40.00)**	**0.0218***
**Modified TNM**<br>**I**<br>**II**<br>**III(IIIA and IIIB)**	**116(63.04)**<br>**58(31.52)**<br>**10(5.44)**	**5(62.50)**<br>**3(37.50)**<br>**0(0)**	**14(35.00)**<br>**13(32.50)**<br>**13(32.50)**	**<0.001***

Hepatocellular carcinoma patients were classified into two groups- “low” and “high”

according to survival receiver–operator characteristic (ROC) curve analysis. The cutting points of Core and ISX separately were 3.5 and 3.0 times of the mRNA expression in HCC tumors than that of the neighboring healthy tissues.SD, standard deviation. Statistical analysis of categorical variables were carried out by one-way ANOVA; *, $p<0.05$

Patients: 232 HCC patients from three medical centers [Chung Ho Memorial Hospital (148HCC), Taiwan Liver Cancer Network (50 HCC) and Changhua Christian Hospital (34 HCC)] were enrolled into the Core and ISX cohort study from May 2017 to May 2022.
